# Reduced Glutathione-Modified Electrode for the Detection of Hydroxyl Free Radicals

**DOI:** 10.3390/bios13020254

**Published:** 2023-02-10

**Authors:** Hamidreza Ghaedamini, Surachet Duanghathaipornsuk, Patrick Onusko, Abdullah M. Binsheheween, Dong-Shik Kim

**Affiliations:** Department of Chemical Engineering, University of Toledo, Toledo, OH 43606, USA

**Keywords:** hydroxyl radicals (•OH), reduced glutathione (GSH), aryl diazonium salt, sensor regeneration, cyclic voltammetry (CV), electrochemical impedance spectroscopy (EIS)

## Abstract

Hydroxyl radicals (•OH) are known as essential chemicals for cells to maintain their normal functions and defensive responses. However, a high concentration of •OH may cause oxidative stress-related diseases, such as cancer, inflammation, and cardiovascular disorders. Therefore, •OH can be used as a biomarker to detect the onset of these disorders at an early stage. Reduced glutathione (GSH), a well-known tripeptide for its antioxidant capacity against reactive oxygen species (ROS), was immobilized on a screen-printed carbon electrode (SPCE) to develop a real-time detection sensor with a high selectivity towards •OH. The signals produced by the interaction of the GSH-modified sensor and •OH were characterized using cyclic voltammetry (CV) and electrochemical impedance spectroscopy (EIS). The CV curve of the GSH-modified sensor in the Fenton reagent exhibited a pair of well-defined peaks, demonstrating the redox reaction of the electrochemical sensor and •OH. The sensor showed a linear relationship between the redox response and the concentration of •OH with a limit of detection (LOD) of 49 µM. Furthermore, using EIS studies, the proposed sensor demonstrated the capability of differentiating •OH from hydrogen peroxide (H_2_O_2_), a similar oxidizing chemical. After being immersed in the Fenton solution for 1 hr, redox peaks in the CV curve of the GSH-modified electrode disappeared, revealing that the immobilized GSH on the electrode was oxidized and turned to glutathione disulfide (GSSG). However, it was demonstrated that the oxidized GSH surface could be reversed back to the reduced state by reacting with a solution of glutathione reductase (GR) and nicotinamide adenine dinucleotide phosphate (NADPH), and possibly reused for •OH detection.

## 1. Introduction

Reactive oxygen species (ROS) are extremely unstable molecules originating from exogenous sources, such as environmental pollution, cigarette smoking, ionizing radiation, and drugs or endogenous sources, such as mitochondrial enzymes and nicotinamide adenine dinucleotide phosphate oxidases (NOXs) [1]. Hydroxyl radical (•OH), hydrogen peroxide (H_2_O_2_), superoxide (•O_2_^−^), alkoxy radical (RO•), peroxyl radical (ROO•), lipid hydroperoxide (LOOH), and ozone (O_3_) are some examples of ROS [2]. ROS are known to be essential molecules for maintaining the proper function of living cells [3,4]. The crucial roles of ROS in living cells include intercellular signaling transduction [5,6,7], the recruitment of immune cells [8,9], activating vital proteins [10,11], and repairing damaged DNA [12]. Even though ROS benefit living cells, an optimum level of ROS is key for maintaining their advantages. In the human body, an imbalance between the generation and removal of ROS leads to cellular oxidative stress, damaging adjacent proteins, lipids, and DNA [13,14,15]. Eventually, a high level of oxidative stress condition leads to developing serious diseases, such as cancer, Alzheimer’s disease, and Parkinson’s disease [16,17,18,19,20].

Among ROS, •OH is the most reactive and destructive species that can damage biomolecules, including carbohydrates, lipids, nucleic acids, and amino acids, posing a significant threat to human beings [1]. Mitochondria are the main organelles in our cells that generate •OH via incompletely reducing molecular oxygen to produce water. The oxygen molecule is initially reduced in the mitochondrial intermembrane to •O_2_^−^, H_2_O_2_, and eventually to •OH [21]. Being extremely toxic, •OH must be detected in order to comprehend the disease etiology and evaluate the efficacy of therapies. However, the identification and measurement of •OH are exceptionally challenging since they have a lifetime of nanoseconds and are highly reactive with adjacent substrates within a short time [22]. Furthermore, due to their extreme chemical reactivity, they can readily damage or destroy the sensing components of detecting devices, preventing them from producing and transducing reliable signals [23,24,25]. Currently, several methods for detecting •OH have been developed, including fluorescence spectroscopy [26], mass spectrometry [27], high-performance liquid chromatography [28], electron spin resonance [29,30], chemiluminescence [31,32], and electrochemical techniques [33]. However, most of these methods have some drawbacks, such as tedious sample preparation, expensive equipment, low sensitivity, and inaccuracy which limit their application [34]. Among different techniques, electrochemical sensing has gained more attention due to its high sensitivity, simple operation, ease of miniaturization, quick response, and low cost [35,36,37]. Although the electrochemical methods have come a long way in detecting •OH, there is still room for developing highly sensitive and selective electrochemical platforms.

Glutathione is a well-known tripeptide with biological activity found in living cells in concentrations between 0.5 mM and 10 mM [38,39]. This sulfhydryl-containing tripeptide serves vital biological roles in living organisms, such as enzyme activity, DNA and protein synthesis, cell protection, and metabolism [40]. Glutathione is often found in its reduced form (GSH), which turns into its oxidized form (GSSG) under oxidative stress [40]. Because of its capacity to donate electrons, GSH has antioxidant and radical scavenging ability with a high affinity toward •OH [41,42,43,44]. A lack of GSH makes the cell susceptible to oxidative damage, and in a wide variety of diseases, including cancer, HIV, and neurological illnesses, a deficiency of GSH is observed [45]. One of the earliest signs of oxidative stress in the human body is a change in GSH concentration or the ratio of GSH to GSSG.

Herein, an electrochemical sensor with high sensitivity and selectivity for rapid detection of •OH is fabricated with a combination of two organic layers deposited on top of a screen-printed carbon electrode (SPCE). An SPCE was used as a transducer because of its cost-effectiveness and compatibility with most materials. Aryl diazonium salt was used to modify the SPCE as a linker to form the second layer consisting of GSH as a sensing element. It is hypothesized that GSH would produce an electrical signal as a result of the redox reaction with •OH, and the signal analyzed with cyclic voltammetry (CV) and electrochemical impedance spectroscopy (EIS) would correspond to the •OH concentration. To the best of our knowledge, there is currently no standard technique capable of generating real-time, accurate, and consistent results for measuring •OH. Therefore, this study would contribute to the development of a standard measuring protocol for •OH. The proposed sensor is expected to be applied in clinical settings for the detection and treatment of disorders resulting from oxidative stress. It could also be utilized in other sectors, such as cosmetic, food, fuel cell, and pharmaceutical industries, where the monitoring and measurement of •OH are required.

The remaining sections of this work are organized as follows. Section 2 outlines the materials and methods used to prepare the sensor and utilize it for detecting •OH generated from the Fenton reaction. The results of the study, including both quantitative and qualitative findings, are discussed in Section 3. This section covers the electrochemical analysis of the sensor, the electrochemical interaction between the sensor and •OH, the effectiveness of the proposed sensor in detecting •OH, and finally, the regeneration of the sensor. The conclusion of the study and recommendations for future research are presented in Section 4.

## 2. Materials and Methods

### 2.1. Materials

L-glutathione reduced (GSH), 4-aminobenzoic acid (ABA), N-hydroxysulfosuccinimide (NHS), N-(3-dimethylaminopropyl)-N′-ethylcarbodiimide hydrochloride (EDC), 2-(N-morpholino)-ethanesulfonic acid (MES), sodium nitrite, hydrochloric acid (HCl), potassium ferrocyanide K_4_[Fe(CN)_6_]·3H_2_O, potassium ferricyanide K_3_[Fe(CN)_6_], iron(II) sulfate heptahydrate (99%), potassium chloride (99%), 30 wt% hydrogen peroxide solution, glutathione reductase from baker’s yeast (*S. cerevisiae*), and nicotinamide adenine dinucleotide phosphate (NADPH) were purchased from Sigma–Aldrich (USA). A screen-printed carbon electrode serving as a transducer for the sensor base was obtained from Pine Instruments, USA. Each electrode consists of a carbon-working electrode with a diameter of 2 mm, a silver/silver chloride reference electrode, and a carbon auxiliary electrode. A Gamry Reference 600 potentiostat (Gamry Instruments, USA) was used to conduct electrochemical impedance spectroscopy (EIS) and cyclic voltammetry (CV) measurements.

### 2.2. Sensor Preparation

**Figure 1:**
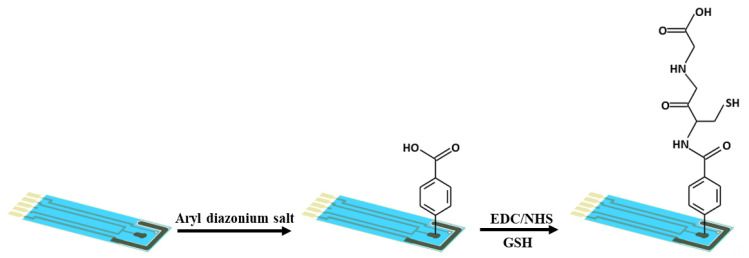
A schematic illustrating the preparation procedure of the GSH-modified electrode.

An aryl diazonium salt solution was prepared by adding 2 mM of sodium nitrite to a solution of 73 mM ABA in 1 mM aqueous HCl, cooled by stirring in an ice bath for 30 min [46]. In order to electrochemically graft aryl diazonium salt on the working electrode, an SPCE was placed in the solution of aryl diazonium salt, and 15 consecutive cycles of CV were performed in the potential range between 0 V and −1 V at a scan rate of 200 mV/s. The functionalized electrodes were rinsed with DI water and methanol to remove physisorbed chemicals. Then, 10 µL of a 100 mM MES buffer solution (pH = 4.5) containing 35 mM NHS and 26 mM EDC was dropped on the aryl diazonium salt-modified electrode to activate the carboxylic groups on the electrode surface for 1 hr. Finally, 10 µL of a 50 mg/mL GSH solution in a 100 mM MES buffer (pH = 4.5) was dropped on the activated aryl diazonium salt-modified electrode to form the GSH layer through covalent bonding with aryl diazonium on the electrode surface. Figure 1 illustrates the fabrication of the GSH-modified electrode.

### 2.3. Detection of •OH Produced by the Fenton Reaction Using the GSH-Modified Electrode

•OH were produced using the Fenton reaction for CV and EIS assays. By mixing equimolar solutions of H_2_O_2_ and FeSO_4_·7H_2_O in equal volumes, the Fenton reaction produces •OH via the reduction of H_2_O_2_ by iron (II) ions. Then, the GSH-modified electrode was placed into the Fenton solution, followed by CV and EIS measurements to determine the interaction between the sensor and •OH. A potential range of −0.2 V to 0.8 V and a scan rate of 100 mV/s were used for the CV tests. The frequency range for EIS experiments was 0.01–10,000 Hz, with AC and DC voltages of 5 mV and 0.23 V, respectively. The H_2_O_2_ solution was shielded with aluminum foil to prevent oxidation through UV light exposure during the experiment. Each measurement was carried out three times for reliability.

## 3. Results and discussion

### 3.1. Electrochemical Characterization of Fabricated Electrodes

#### 3.1.1. Cyclic Voltammetry

**Figure 2:**
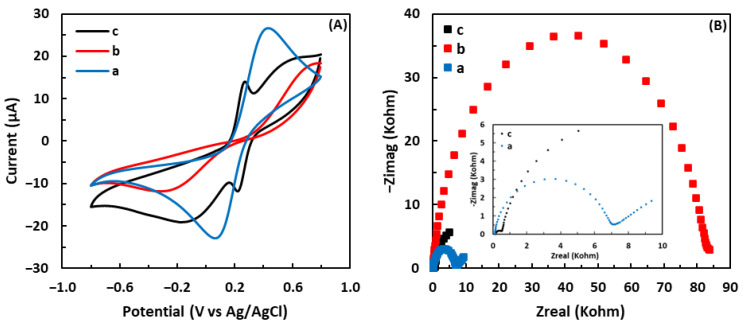
(**A**) Cyclic voltammograms and (**B**) Nyquist plots for (a) the bare electrode, (b) the aryl diazonium-modified electrode, and (c) the GSH-modified electrode, in 5 mM solution of [Fe(CN)_6_]^3−/4−^ containing 0.1 M KCl.

The electrochemical behavior of the electrodes was characterized at each functionalization step by performing CV and EIS in 0.1 M phosphate buffer solution (PBS, pH 7.2) containing 5 mM [Fe(CN)_6_]^3−/4−^ as the redox probe and 0.1 M KCl to verify the success of surface modification [47]. CV tests were performed in the potential range of −0.8 V to 0.8 V and a scan rate of 100 mV/s. For cyclic voltammograms in Figure 2A, the blue curve represents the current response of the bare electrode, which drastically decreases upon modification with aryl diazonium salt (red curve), demonstrating the formation of aryl diazonium salt. After the electrodeposition of aryl diazonium salt, the electrical current significantly decreased due to the presence of a non-conductive layer of aryl diazonium salt on the electrode surface. Aryl diazonium salt reduces the rate of electron transfer between the electrolyte solution and electrode surface, causing a significant drop in the current [48]. After the introduction of GSH, an obvious pair of well-defined redox peaks appeared in the cyclic voltammogram of the GSH-modified electrode (black curve). These peaks are ascribed to the redox of GSH on the electrode, confirming that the electrode has been successfully modified with GSH. The redox peak potential difference of the GSH-modified electrode was significantly lower than that of the bare electrode, demonstrating the electrical catalytic and conductive properties of the GSH-modified electrode [49].

#### 3.1.2. Electrochemical Impedance Spectroscopy

Additionally, EIS was used to confirm the successful immobilization of the first and second layers of aryl diazonium salt and GSH, respectively. The frequency range was from 0.1 Hz to 100,000 Hz, and the AC and DC voltages were 5 mV and 0.23 V, respectively. Figure 2B shows the EIS results of the bare electrode, the aryl diazonium-modified electrode, and the GSH-modified electrode. In Nyquist plots, a linear section at lower frequencies is attributed to a process limited by diffusion, whereas a semicircle part at higher frequencies corresponds to a process limited by electron transfer. Moreover, the diameter of the semicircle at higher frequencies reflects the interfacial electron-transfer resistance (R_et_) [50,51]. After modification with the aryl diazonium salt, R_et_ significantly increased (red curve) compared to the bare electrode (blue curve). It is noted that the aryl diazonium-modified electrode showed the largest diameter where the organic salt deposited on the electrode inhibited the electron transfer, increasing the electrode resistance. Immobilizing GSH on top of the aryl diazonium layer resulted in the smallest semicircle diameter (black curve), i.e., the lowest R_et_, which is associated with the inherent redox reactivity of GSH [52]. It is also noted that the aryl diazonium-modified electrode showed no diffusion-limited process.

### 3.2. Detection of •OH in the Fenton Solution by the GSH-Modified Electrode

**Figure 3:**
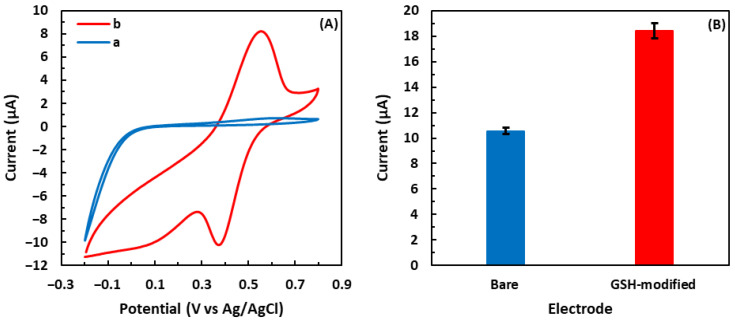
(**A**) Cyclic voltammograms of (a) the bare electrode and (b) the GSH-modified electrode in 5 mM •OH. (**B**) Redox responses of the bare electrode and the GSH-modified electrode to 5 mM •OH. The error bars indicate the standard deviation of three repetitive experiments.

The detection of •OH in the Fenton solution was carried out using the GSH-modified electrode. Figure 3A indicates the CV curves of the bare electrode and GSH-modified electrode in 5 mM •OH. The CV curve of the bare electrode showed no redox peaks, demonstrating that there is no redox reaction between the bare electrode and •OH. On the other hand, a pair of well-defined redox peaks associated with the redox reaction of the GSH surface and •OH was clearly observed in the curve of the GSH-modified electrode. The sensor response to •OH was measured using the current difference between the reduction and oxidation peaks. As illustrated in Figure 3B, the electrode modified with GSH demonstrated a 75% greater response to 5 mM •OH (18.4 µA) than the bare electrode (10.5 µA). 

**Figure 4:**
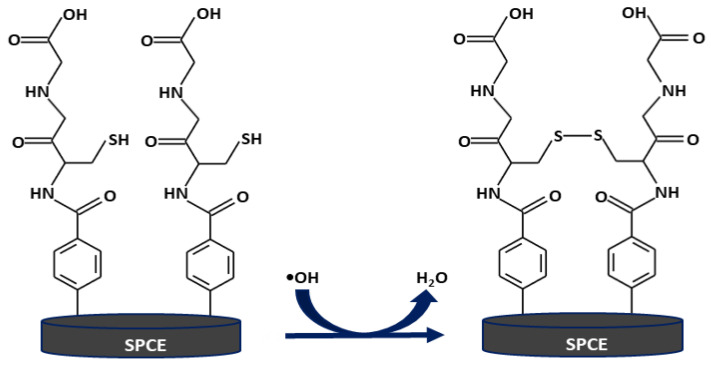
A schematic illustrating the oxidation reaction of the GSH-modified electrode with •OH.

The reaction between the GSH surface and •OH is depicted in Figure 4. •OH attack the thiol groups of the GSH-modified electrode and grabs their hydrogen atoms, stabilizing themselves to water molecules, leading to the GSH transformation to corresponding disulfide GSSG on the electrode. 

### 3.3. Effect of the Scan Rate on the GSH-Modified Electrode

**Figure 5:**
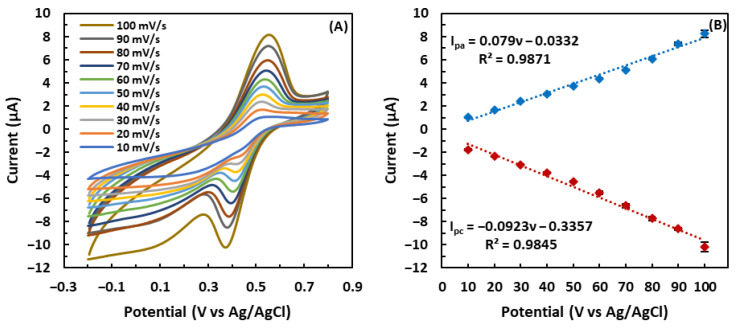
(**A**) Cyclic voltammograms of the GSH-modified electrode at scan rates from 10 mV/s to 100 mV/s in 5 mM •OH. (**B**) Relationship of the peak current response and the scan rate (ν). The error bars indicate the standard deviation of three repetitive experiments.

To investigate the electrochemical reaction kinetics, the CV response of the GSH-modified electrode to 5 mM •OH was studied by varying the scan rate (ν) from 10 mV/s to 100 mV/s. As shown in Figure 5A, raising the scan rate led to a gradual increase in both anodic and cathodic peak currents. Figure 5B illustrates that the peak current response of the GSH-modified electrode exhibited a linear relationship with the scan rate, demonstrating that the electron transfer between GSH and the electrode is a classical surface-controlled electrochemical process [53]. Figure 5A reveals that as the scan rate increased, the cathodic and anodic peak potentials gradually shifted toward negative and positive values, respectively, i.e., the peak-to-peak separation expanded as the scan rate increased. Moreover, the anodic peak current change amount (∆I_pa)_ of the GSH-modified electrode was not equal to the cathodic peak current change amount (∆I_pc)_. These results demonstrate that the redox reaction is a quasi-reversible process [54,55]. The surface coverage of GSH, Γ, on the electrode was estimated according to Equation (1) [56]:

Γ = Q/nFA(1)

where the charge consumed by GSH, denoted by Q, is the area of the GSH oxidation peak, n is the number of transferred electrons involved in GSH oxidation (n = 1), F represents the Faraday constant, and A is the area of the working electrode. Q was determined as 13.3 µC using the cyclic voltammogram at a scanning speed of 100 mV/s. Based on the known parameters in Equation (1), Γ was found as 4.38 × 10^−9^ mol cm^−2^. This considerable surface coverage guarantees high electrochemical signal sensitivity [57].

### 3.4. Calibration Curve

**Figure 6:**
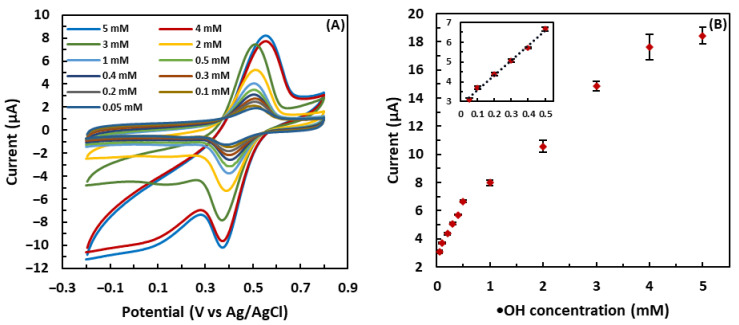
(**A**) Cyclic voltammograms of the GSH-modified electrode for •OH with different concentrations from 0.05 mM to 5 mM. (**B**) Relationship of the redox response (∆A) with •OH concentration. The error bars indicate the standard deviation of three repetitive experiments.

CV analysis was performed to investigate the response of the GSH-modified electrode to different concentrations of •OH generated by the Fenton reaction. As shown in Figure 6, the redox response of the GSH-modified electrode increased along with the •OH concentration ranging from 0.05 mM to 5 mM, although the trend of increase at low concentrations was different from that at high concentrations. As for the concentration range from 0.05 mM to 0.5 mM, the sensor exhibited a linear increase in the current change with the increasing •OH concentration (R^2^ = 0.99). However, at high concentrations from 0.5 mM to 5 mM, the relationship between the current change and •OH concentration deviated from linearity. The sensor responded to •OH with a higher sensitivity in the concentration range from 0.05 mM to 0.5 mM, as the slope of the regression line in this region was greater than that in the region of high concentrations. Above 0.5 mM, the increasing rate of the current slowed down and then eventually leveled off around 18.4 µA, most likely due to the insufficient GSH on the electrode to scavenge and detect a high concentration of •OH. In other words, when the •OH concentration was higher than 4 mM, the sensor response plateaued as all the GSH was oxidized with 4 mM •OH. The linear relationship between the redox response and •OH concentration between 0.05 mM and 0.5 mM was used to determine the limit of detection (LOD) of the GSH-modified sensor. Equation 3.3 × SD/b was applied to the data in the linear region, where SD represents the standard deviation of the blank, and b is the slope of the regression line [58]. The LOD of the proposed sensor was calculated as 49 µM, which is comparable to many previously reported electrochemical sensors for the detection of •OH [1,34,59,60]. As the normal concentration of •OH in the blood of healthy people is between 200 and 400 mM, and higher in case of oxidative stress conditions [61], the proposed sensor can be used to detect •OH in blood serum.

### 3.5. Selectivity and Stability of the GSH-Modified Sensor

**Figure 7:**
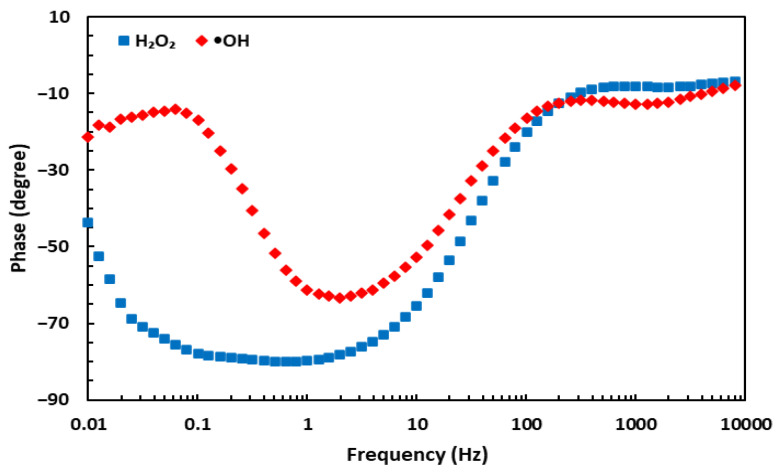
Bode plots for phase angle shifts for the EIS results obtained with the GSH-modified electrode in the presence of •OH and H_2_O_2_.

Selectivity is one of the most crucial analytic characteristics of sensors. EIS was used to examine the selectivity of the GSH-modified sensor. Figure 7 demonstrates that the proposed sensor can distinguish between •OH generated in the Fenton reaction and a similar oxidizing compound, such as H_2_O_2_. The pattern of phase angle shift for •OH in a Bode plot differs substantially from that for H_2_O_2_. Therefore, not only is the GSH-modified sensor highly sensitive, but also it is capable of differentiating between •OH and other similar ROS. In more detail, the phase angle shifts for •OH occurred at 0.1 and 3.16 Hz, whereas only a change at 1.27 Hz was observed for H_2_O_2_. Additionally, the stability of the electrochemical sensor was examined by storing the GSH-modified electrode at 4 °C for a week. The redox response of the sensor showed no significant change, revealing the satisfactory stability of the proposed sensor. 

### 3.6. Regeneration of the Sensor

**Figure 8:**
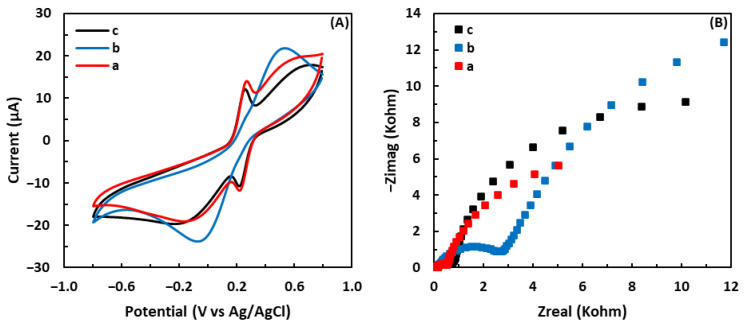
(**A**) Cyclic voltammograms and (**B**) Nyquist plots of (a) the GSH-modified electrode before being treated with the Fenton reagent, (b) after being treated with the Fenton reagent, and (c) after regenerated by NADPH and GR in 5 mM solution of [Fe(CN)_6_]^3−/4−^ containing 0.1 M KCl.

CV and EIS tests were employed to investigate the oxidation of the GSH surface after the sensor was exposed to •OH. First, the freshly GSH-modified electrode was immersed in the electrolyte solution, as mentioned in Section 3.1, and CV and EIS assays were performed. The electrode was then transferred to the Fenton solution for 1 hr. Subsequently, the oxidized GSH-modified electrode was again transferred to the electrolyte solution and analyzed by CV and EIS. As shown in Figure 8A, a pair of well-defined peaks associated with the redox reaction of GSH disappeared in the CV curve of the sensor after being exposed to •OH. In addition, Figure 8B shows that the diameter of the semicircle increased after the GSH-modified electrode was immersed in the Fenton reagent, demonstrating the increase in R_et_. These results demonstrate that after being exposed to •OH, the immobilized GSH on the electrode is oxidized and turned into GSSG by •OH.

It is reported that the GSSG solution can be reduced back to GSH using glutathione reductase (GR) in conjunction with nicotinamide adenine dinucleotide phosphate (NADPH) [62,63,64]. To evaluate if the GSH surface could be restored, 10 µL of a solution composed of 1 mM NADPH and GR (0.5 unit/mL) in PBS (pH = 7) was dropped on the oxidized GSH-modified electrode and allowed to react for 2 h. After regeneration by GR and NADPH, a pair of redox peaks reappeared in the CV curve of the regenerated sensor, as shown in Figure 8A. Moreover, the Nyquist plot of the regenerated sensor reveals that the diameter of the semicircle decreased after exposure to GR and NADPH, demonstrating that the sensor regained its electrical catalytic and conductive properties. Comparing the peak current response of the fresh sensor with that of the regenerated sensor, the percentage of restoration was calculated as 84%.

## 4. Conclusions and Future Work

The GSH-modified electrode for the detection of •OH was prepared with the electrochemical deposition method using the aryl diazonium salt as a linker. The redox reaction between the electrochemical sensor and •OH was demonstrated by the appearance of two well-defined peaks on the CV curve of the sensor after modification with GSH. The GSH-modified sensor exhibited a linear relationship between •OH concentration and the current change in the range of 0.05 mM to 0.5 mM •OH and a LOD of 49 µM. In terms of selectivity, the proposed sensor demonstrated the ability to differentiate between •OH and a similar oxidizing chemical, such as H_2_O_2_, which is crucial for its application in complex systems. Furthermore, electron transfer between immobilized GSH and the electrode was found to be a classical surface-controlled electrochemical process. After exposure to •OH, the immobilized GSH on the electrode was oxidized and converted to GSSG. However, the oxidized GSH-modified sensor demonstrated the capacity to be regenerated using GR and NADPH. In detail, 84% of oxidized GSH could be converted back to the reduced GSH. Given the high sensitivity and specificity of the GSH-modified sensor for detecting •OH, future research could focus on using the sensor for in vitro detection of •OH in biological samples, such as animal cells and body fluids, including blood, plasma, urine, and cerebrospinal fluid. Another perspective is to reduce the sensor size for in vivo detection.

## Data Availability

Not applicable.
